# Complex Event Processing for Sensor Stream Data

**DOI:** 10.3390/s18093084

**Published:** 2018-09-13

**Authors:** Kyoungsoo Bok, Daeyun Kim, Jaesoo Yoo

**Affiliations:** 1 Department of Information and Communication Engineering, Chungbuk National University, Chungdae-ro 1, Seowon-Gu, Cheongju, Chungbuk 28644, Korea; ksbok@chungbuk.ac.kr; 2 Department of Big Data, Chungbuk National University, Chungdae-ro 1, Seowon-Gu, Cheongju, Chungbuk 28644, Korea; kdy0573@chungbuk.ac.kr

**Keywords:** complex event, similar operation, redundant operation, query tree, event detection

## Abstract

As a large amount of stream data are generated through sensors over the Internet of Things environment, studies on complex event processing have been conducted to detect information required by users or specific applications in real time. A complex event is made by combining primitive events through a number of operators. However, the existing complex event-processing methods take a long time because they do not consider similarity and redundancy of operators. In this paper, we propose a new complex event-processing method considering similar and redundant operations for stream data from sensors in real time. In the proposed method, a similar operation in common events is converted into a virtual operator, and redundant operations on the same events are converted into a single operator. The event query tree for complex event detection is reconstructed using the converted operators. Through this method, the cost of comparison and inspection of similar and redundant operations is reduced, thereby decreasing the overall processing cost. To prove the superior performance of the proposed method, its performance is evaluated in comparison with existing methods.

## 1. Introduction

Due to the trend of miniaturization and price drop of communication modules, communication among objects in daily life is now possible. Along with this trend, environments where things can create and share information are provided due to the penetration of intelligent digital devices. As things such as vehicles, refrigerators, bicycles and even shoes are now able to communicate, new information technology services are emerging. The Internet of Things (IoT) is intelligent networking where a variety of things are connected to wired and wireless networks and collect and share information for interaction [1,2,3]. Through the IoT, information can be created, collected and shared organically, as everything including people, surrounding things and data are connected through the network. 

Sensor network technology is essential to create, collect and exchange information for things over the IoT environment [4,5,6]. Physical states and surrounding environment information are collected by attaching sensors to various things, and information is transmitted among things using the sensor network technology. A technology that can process or analyze big data collected through the sensor network technology is required to provide intelligent services in the IoT environment [7,8,9,10,11]. In the IoT environment, sensor networks can be used to determine future predictions and trends by detecting physical situations or analyzing collected information. Recently, time-critical applications have been developing to analyze data sensing from multiple sources in order to respond in real time in case of abnormal and dangerous situations in the areas of disaster safety, manufacturing, transportation and communication [12,13,14]. For example, manufacturers are building smart factories that collect and analyze product production data, environmental data and so on to determine if defective products have occurred or to detect hazardous situations in a factory. In transportation, intelligent traffic services are being developed to recognize situations around vehicles and roads through sensors and to provide vehicles and pedestrians with a risk alert service in the case of a hazardous or sudden event.

A large number of studies have been conducted to process or analyze stream data generated through numerous sensors in real time [15,16,17,18]. In particular, a complex event processing (CEP) method has been developed to remove unnecessary information from a large amount of stream data generated through sensors or to detect information that is required only for a specific application [19,20,21]. Generally, data processing is conducted by storing collected data in disks followed by batch processing to create needed information. Thus, real-time processing is not appropriate since processing delay occurs if a large amount of stream data is generated through sensors [22,23,24]. CEP analyzes real-time stream data that are generated continuously, thereby delivering required information selectively once users or application requirements are inputted as a continuous query form [25,26,27]. CEP utilizes in-memory-based processing and event-driven architecture (EDA) to process stream data in real time generated from sensor networks [28,29,30].

In CEP, stream data generated through sensors are defined as primitive events, which are then combined through various operators to detect meaningful events to users or applications. In stream-based and shared event (SASE) processing, complex events are expressed in the automata form, and complex events are stored in a stack as primitive events and connected by pointers based on the occurrence time [31,32]. However, complex events consisting of the same primitive events are processed redundantly, which increases processing time. In the Radio frequency identification (RFID) complex event detection algorithm (RCEDA), a pattern of complex events is configured as a graph form, and all primitive events are delivered to parent nodes of the graph, thereby performing an operation that compares the conditions [33]. However, if primitive events that require similar operations are introduced, the operation for each event is processed individually, thereby increasing computational load. Siddhi can reduce processing amount, since it can process redundant operations into a single operation using a pipeline format. However, it has a problem of increasing computational load as well due to individual processing of a large number of similar operations. 

This paper presents an efficient CEP method considering similar and redundant operations on sensor stream data. The proposed method generates an event query tree and detects similar and redundant operations by circulating the tree to detect complex events, which is similar to the structure of RCEDA. The similar operations that perform similar comparison operations or operations that are interrelated with common events are combined by a single virtual operator for processing. The redundant operations (i.e., those that perform the same operations on the same events) are processed by a single operation. Once a primitive event occurs, the hash table is searched and the primitive event is delivered to the event query tree to detect a complex event. Since a virtual operator expresses a number of operations that perform similar operations, it expresses event occurrences through bits, and when events that satisfy the condition occur, the events are delivered to the parent node for the next operation. 

This paper is organized as follows. In Section 2, related study trends and issues are described. In Section 3, the proposed method is described in detail, and in Section 4, performance evaluation is conducted to prove the superiority of the proposed method. Finally, in Section 5, conclusions and future research are presented.

## 2. Related Works

RFID is a technology used to identify and track objects in logistics and supply chain management, retail, healthcare, factory automation and security [34,35,36]. The objects with attached RFID tags are automatically identified by RFID readers. The RFID reader sends energy via RF signal to the tag, and the tag sends back a modulated signal. RFID readers can sense a number of RFID tags at the same time. RFID uses short-range radio technology to transfer data between the readers and tags. Since RFID streams can be continuously generated, CEP is required to detect meaningful events in RFID streams. RFID events are divided into primitive events and complex events in CEP. A primitive event is an RFID tag sensed by an RFID reader. A complex event is a composite event which consists of primitive events for applications or users [33,37]. 

SASE is a method to filter RFID data, monitor interrelated patterns, and detect meaningful information [31,32]. SASE consists of event patterns of complex events, conditions (where), and time intervals (within). The event pattern is a combination of primitive events, which are combined through AND, OR, NOT, and SEQ operators. To select events, sequence scan and construction (SSC), selection, within, and negation (NG) operations are performed. The SSC operation configures events in the automata form, and stores occurring events in the stack through sequential scan. A state of automata changes according to the sequential occurrence of primitive events. In SASE, even the operations consisting of the same primitive events are processed individually. Thus, it has a problem of increased overall computational load due to redundant inspection when processing similar or redundant operations. 

RCEDA is a RFID complex event-processing method using a graph-based computation model. RCEDA presents the definition rules and processing method to process complex events efficiently [33]. RCEDA employs primitive operations such as AND, OR and NOT, and additional operators such as SEQ, TSEQ, SEQ+ and TSEQ+ to configure complex events. The complex event is constructed by combining operator trees and primitive events. In the leaf node of the tree, a primitive event is positioned, and in the non-leaf node, an operator that connects events is positioned. Once the event in the leaf node matches the condition, it is transferred to an operator in the parent node, and if the event matches the condition of the operation in the root node, it is detected as a complex event. In the case of the NOT operation, an event cannot be detected in the corresponding operator. Thus, a virtual node is made in the upper hierarchy, and if the condition in the virtual node is satisfied in the NOT operator in the lower hierarchy, the event is delivered to the parent node. In RCEDA, complex events are registered as a graph form, and all occurred primitive events are delivered to the parent node, thereby performing the complex event processing. 

Siddhi consists of operations connected through the event queue, and operations are processed through the pipeline mode [38]. The system consists of an event queue that stores data, and processors that process input events. Once the primitive event is received through the input adapter, the model is changed into the common data model. Each processor consists of executors, and each executor processes the received events. Once the event that matches the condition occurs, the next step is performed through the pipeline processing using the publication/subscription model. Siddhi uses similar queries employed in the SQL, and constructs a query tree followed by performing optimization. In the Siddhi system, redundant subqueries are processed simultaneously to reduce the computational load. 

The bitmap index-based CEP method processes complex events using a trigger according to whether events occur or not [37]. In the above method, even if all events that make up the complex events do not occur, operations continue for partially occurred events, thereby performing a large number of operations and consuming a large amount of memory space. To solve this problem, the bitmap index-based CEP method does not process all events that have occurred in a large amount of stream data, but complex events are processed only when events that correspond to complex events registered in the application layer occur. However, this has the problem that computational load is not reduced significantly, since no consideration is given to operators at all in the case of complex events consisting of the same primitive events. In [37], the complex events is detected only when the minimum conditions for processing of events with regard to RFID stream data are satisfied. To inspect the minimum conditions of events, complex events are registered in the grid index, and they are extracted using the query indexes and bitmap. It only compares whether primitive events that make up the complex events occur or not, and when all primitive events that make up the complex events occur, the event detection operation is performed. However, since complex events, which have similar operations, have redundant searches for events stored in the bitmap, the problem of increased computational load is incurred. 

Since the existing methods cannot process events by sharing similar operations as one, they have a drawback of increasing computation due to individual processing of events that have operations of similar patterns. They also have no consideration about redundant operations that have the same primitive events. Due to this drawback, computations to process complex events from a voluminous data stream are increased significantly, and completion time is delayed because events cannot be processed in a given time. Thus, a study on reduction of overall computational load is needed in consideration of cases where primitive events that make up the complex events have similar and redundant operations.

## 3. Complex Event Processing

### 3.1. Overall Procedure

The existing CEP methods require a large amount of comparison and inspection cost, since they individually process similar and redundant operations that process the same event when a large amount of stream data is generated in real time through sensors. Thus, a study on processing a large amount of complex events rapidly is needed for reducing the computational load of complex event detection. The proposed method is a new CEP method that reduces the processing cost of similar and redundant operations. In this method, the execution plan for the detection of complex events is converted into a tree, and an event query tree is reconstructed with regard to similar and redundant operations. Similar operations are expressed as a single virtual operator to determine whether the event occurs or not. 

**Figure 1:**
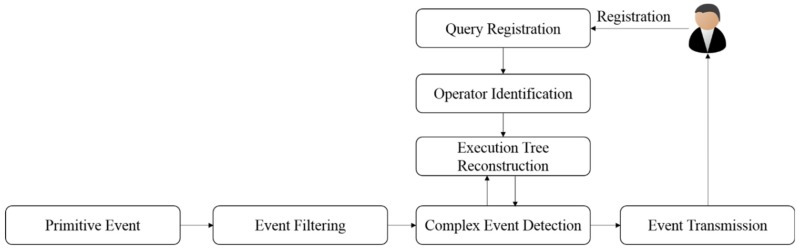
Overall complex event processing procedure.

Figure 1 shows the overall processing procedure for complex events. Once a user or specific application registers a query to detect a complex event, an event query tree is created to process the event. In the operator identification, the event query tree is traversed to determine whether the event is similar or redundant. The similar operation is changed into a single virtual operator, and the redundant operation is converted into a single operation to reconstruct the event query tree. Once a primitive event occurs through sensors, errors or redundant data are filtered. The complex event detection determines whether the primitive event satisfies the complex event condition after event filtering. Here, the reconstructed event query tree is employed to determine whether the complex event condition is met. Once the complex event is detected, the event information is transmitted to the user.

### 3.2. Complex Event Registration

The input stream collected through sensors in the IoT environment is defined as a primitive event, and a complex event is inferred by a query requested from a specific user and application to detect meaningful information or remove unnecessary information. The primitive event is expressed as raw data collected through sensors at a specific period of time, which is represented as <e,t,v>. Here, e refers to a sensor identifier as a primitive event type, t refers to a time that collected the primitive event, and v refers to a value collected through the sensor. The primitive event means the original data collected through the sensor itself. Each data collected by each sensor is defined as a primary event and used as input data for complex event processing. Complex events are generated by a combination of primitive events to create or extract meaningful information needed by a user or application. Various event operators are used to generate composite events in combination with primitive events. For the event operator, the operator defined in RCEDA is used in this study to define the complex event. 

**Figure 2:**
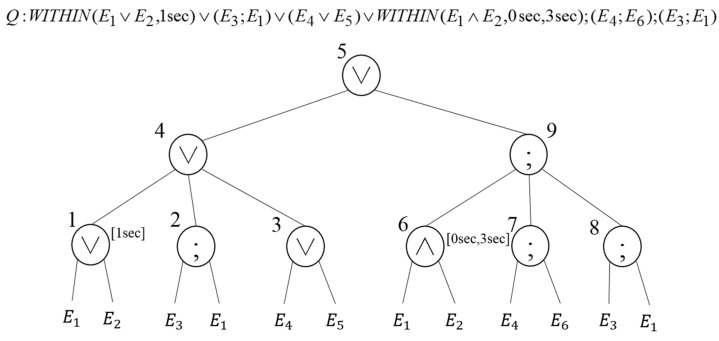
Complex event query tree.

A user and application registers a complex event that combines primitive events generated in real time through sensors as a type of continuous query. The event query tree is created to detect whether the registered complex event occurs or not. The event query tree consists of the combination of primitive event and operator, which is similar to that of RCEDA. A primitive event is stored in the leaf node in the tree, and operators for event combination or combined events are stored in the internal node. Once the event query tree is created, the tree is in-order traversed to detect similar and redundant operations, thereby creating a complex event information table (CEIT). The CEIT stores the operator’s address, operation type, and parent node address. Figure 2 shows the event query tree of complex event Q. When the event query tree is created, the tree is in-order traversed and numbers one to nine are assigned according to the visit order of the internal node. In addition, information about the visited node is stored in the CEIT. 

### 3.3. Operator Identification

Since a number of operators are contained in a complex event, the comparison and inspection costs increase if similar and redundant operations are processed individually. To solve this problem, similar and redundant operations are detected and their operators are converted. In this paper, a redundant operation is defined when the number of the same operators is one or more in the same event, and a similar operation is defined when interrelated operations or similar comparison operations are performed with regard to the same event. 

Table 1 presents a type of similar operations. OA refers to a case where OR and AND operations are processed simultaneously with regard to the same event; SS refers to a case where the earlier-occurred operation performs the same two SEQ operations; and OS refers to a case where the earlier-occurred event in the SEQ operation performs an OR operation. In the OA operation, when one of the events consisting of OR operations occurs, the event is transmitted to the higher node. In contrast, when all events in the AND operation occur, the events are transmitted to the parent node. That is, when one of the events that participates in OR and AND operations occurs, the event occurrence is transmitted to the parent node in the case of OR operation and the occurrence of the previous event is set by a bit. In the case of AND operation, only when related other events occur is the result transmitted to the parent node for processing. Since the SEQ operation takes the occurrence order of events into account, the result of SS is delivered to the parent node depending on whether the other event occurs or not while the one event that shall occur first between two SEQ events has already occurred in the node. The result of the OR operation is transferred to the parent node if the precedent event in the SEQ operation is one of the events that should be processed in the OR operation, and the result of the SEQ operation is delivered to the parent node if the event that participated in the SEQ operation occurs. 

The similar and redundant operations are detected while performing in-order traversal over the event query tree. Here, the CEIT created in the complex event registration process is used. Table 2 presents the similar operation detection results, and Table 3 presents the redundant operation detection results. OA and OS are detected as similar operations when there is an event query tree shown in Figure 2. The AND and OR operations that can be combined as the similar operation OA, and OR and SEQ operations that can be combined as the similar operation OS, are detected. As presented in Table 3, the same operation on $E_{1}$ and $E_{3}$ events is performed in Nodes 2 and 8, and it is detected as the redundant operation. 

### 3.4. Reconstruction of Event Query Tree 

The event query tree is created to detect complex events once they are registered. The previously created event query tree is reconstructed based on the similar and redundant operation detection results to reduce the processing cost. In the event query tree, similar and redundant operations are converted into a single operator to reduce the comparison and inspection cost. Similar operations are converted into a virtual operator, and redundant operations are represented by a single operator. A virtual operator is expressed in the lowest node number among the unique numbers in the nodes related to the similar operations. In addition, the Event Occurrence Information Table (EOIT) is created to determine whether a related event occurs or not in the virtual operator where two or more operations are combined. In the EOIT, the Occurrence Bit (OB), which represents an operation to be performed according to the event occurrence, and Parent node Address (PA), where the next operation process is performed, are stored. 

**Figure 3:**
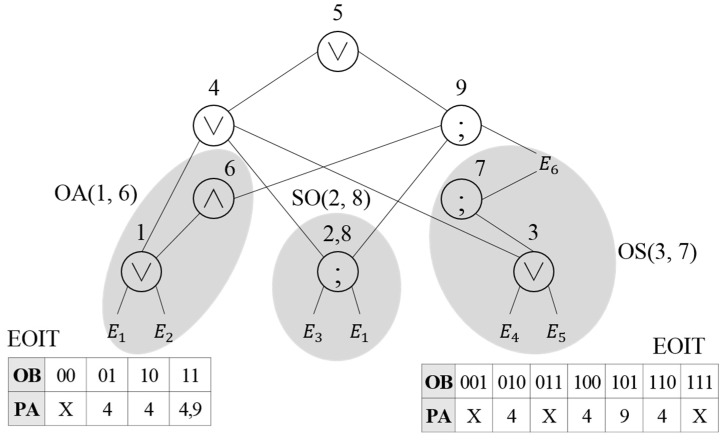
Reconstruction of the event query tree using virtual operation.

Figure 3 shows the reconstructed results of the event query tree shown in Figure 2. The similar operations are processed with a single virtual operator to reduce the comparison operation cost during event detection. In the node that represents the virtual operator, the EOIT is stored together, and the number of bits equal to the number of primitive events related to the virtual operator is assigned to the EOIT. Since the number of primitive events of operations in the virtual operation OA(1,6) is two, the bit that represents the occurrence is set to two, and the address of the higher node where the next operation will be performed is stored. Since the virtual operation OS(3,7) is related to three events, the bit that represents the occurrence is set to three. Nodes 2 and 8 are redundant operations where the same SEQ operation is performed in $E_{1}$ and $E_{3}$. To reduce the processing cost of the redundant operations, the operation of Nodes 2 and 8 is expressed as SO(2,8). 

**Figure 4:**
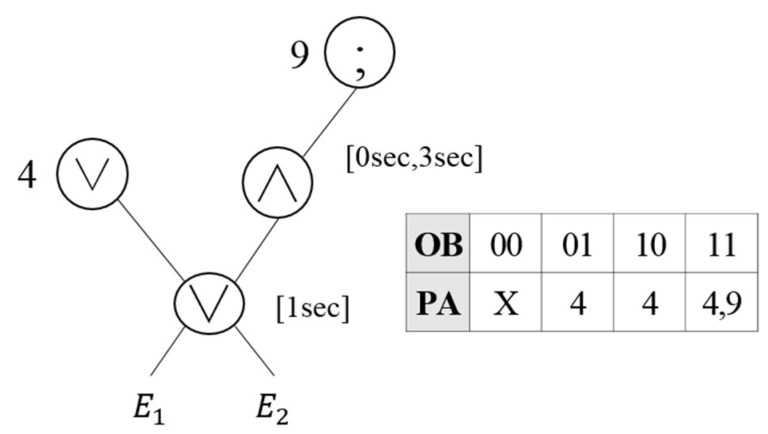
Creation of virtual operator OA(1,6).

As presented in Table 2, since Nodes 1 and 6 perform the OR and AND operations on the same events $E_{1}$ and $E_{2}$, virtual operator OA(1,6) is created. Figure 4 shows the reconstructed results of the event query tree through the virtual operator OA(1,6). The operation of Node 1 in the event query tree is modified to OA(1,6), and the operation of Node 6 is deleted. Once the event is inputted, the event occurrence is expressed by the bit in the OB, and when the event that matches the operation occurs, the next execution request is given to the parent node address. Once $E_{1}$ and $E_{2}$ occur, the result of the OR operation is delivered to the parent node. If other events occur in addition to the event that satisfies the OR operation, the AND operation condition is checked and the next execution request is given to all the parent nodes of the OR and AND operations. 

**Figure 5:**
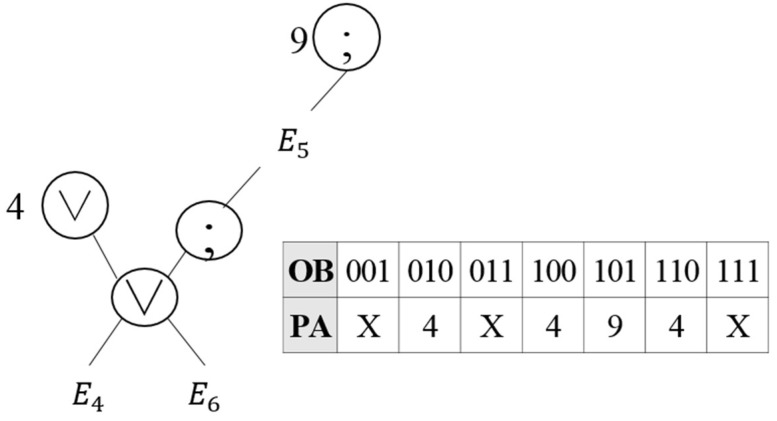
Creation of virtual operator OS(3,7).

In the similar operation OS(3,7), as presented in Table 2, in the SEQ operation in Node 3 and the OR operation in Node 7, the precedent event in the SEQ operation is included in the OR operation. Thus, the SEQ operation in Node 3 and the OR operation in Node 7 can be combined to represent OS(3,7), as shown in Figure 5. The virtual operator OS(3,7) is stored in Node 3, whose number is lower, and Node 7 is deleted. Since events $E_{4}$, $E_{5}$ and $E_{6}$ are related in the virtual operator OS(3,7), three bits are assigned to the OB of the EOIT, and once an event that matches the operation condition occurs, the OB is checked before the transfer to the parent node. 

**Figure 6:**
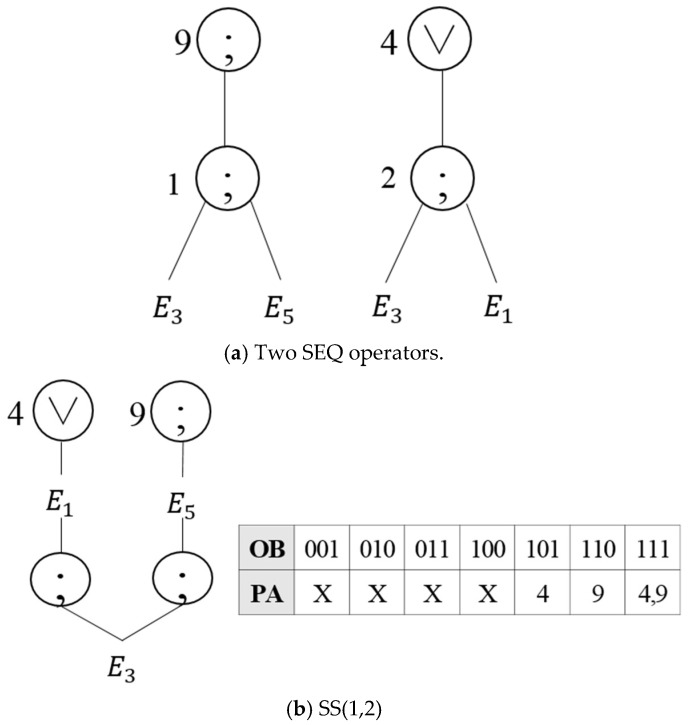
Creation of virtual operator SS(1,2).

The SS operation is related to two SEQ operations. Assuming that two SEQ operations are $E_{3};E_{5}$ and $E_{3};E_{1}$, then $E_{3}$ is an operation that shall occur first in both of the two SEQ operations as shown in Figure 6a. Thus, virtual operator SS(1,2) is created for the two SEQ operations, as shown in Figure 6b. If $E_{3}$ occurs first followed by $E_{1}$ or $E_{5}$, then the next execution request is given to the parent node stored in the PA of the EOIT. 

**Figure 7:**
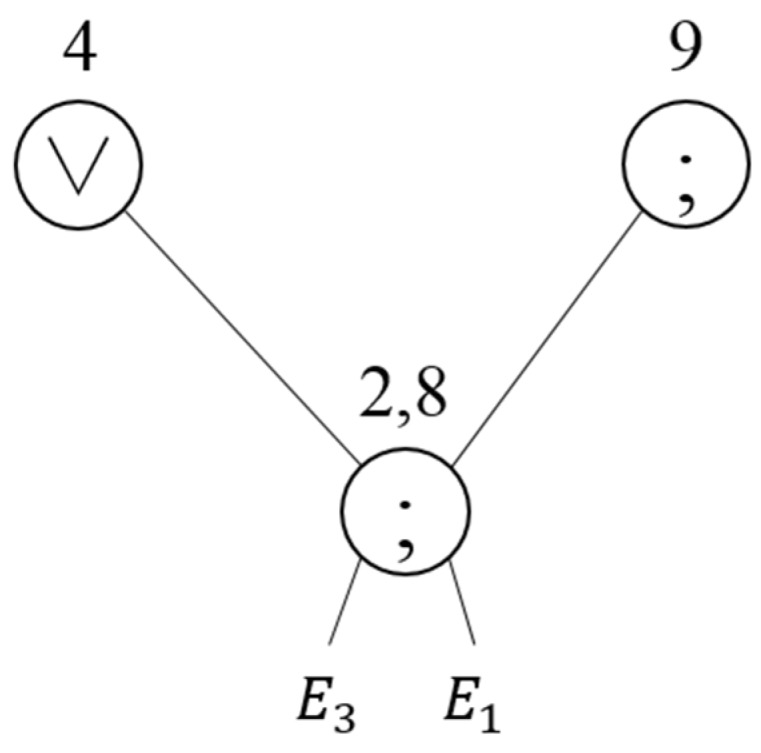
Complex query tree that combines redundant operations.

Since the same operation is performed with regard to the same events in the redundant operations, the event query tree is restructured to perform only a single operation for two operations. If the query is modified to perform a single operation for the redundant operations, the next execution operation should be processed individually. For example, although Nodes 2 and 8 are redundant operations in Table 3, the execution operation that follows Node 2 is different from that following Node 8. Thus, addresses of all nodes where the next operation will be performed should be stored. Nodes 2 and 8 in Table 3 are redundant operations where the SEQ operation is performed with regard to $E_{1}$ and $E_{3}$. SO(2,8) is created for the redundant operation, as shown in Figure 7. Nodes 2 and 8, where the redundant operation is performed, are expressed as a single node, and the information of parent nodes of Nodes 2 and 8 is added to perform the next operations individually. 

### 3.5. Complex Event Detection

After the event query tree is reconstructed using the similar and redundant operations, complex events are detected through continuous comparison between the input stream data and query tree. The general complex event detection process is similar to that of RCEDA except for the processing procedure for similar and redundant operations. Once primitive events are generated through sensors, the hash table is searched to deliver the primitive events to the event query tree where the operations are performed for the primitive events. If the conditions performed in the event query tree are general operators, all processes are the same as those done in RCEDA. If the operation to be performed is a virtual operator, the event occurrence is set up, and when an event that satisfies the condition occurs, the next execution request is given to the parent node stored in the PA of the EOIT. If the operation to be performed is a redundant operation, the next execution request is given to multiple parent nodes after the current operation. 

**Figure 8:**
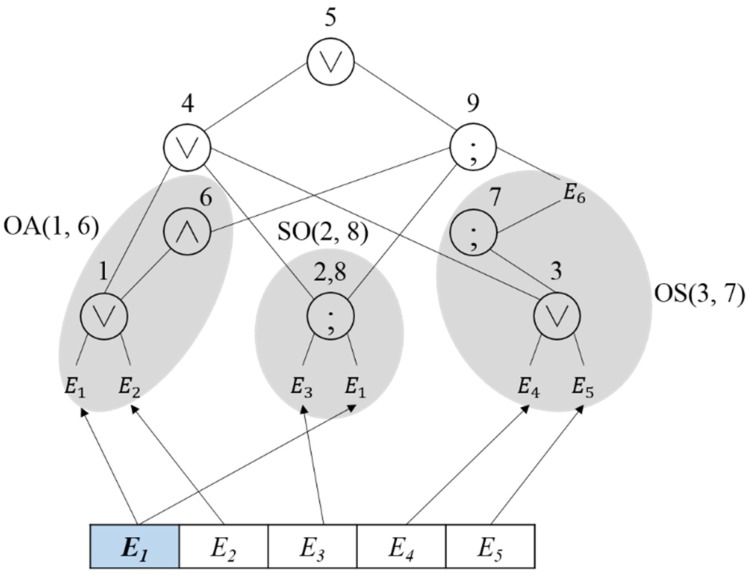
Hash table.

When a primitive event occurs, the location of the event query tree where the operation will be performed with regard to the primitive event should be identified. To do so, a hash table is created. The hash table is composed of a list of the addresses of the event query tree where the operation is performed with regard to the primitive events. Once a primitive event occurs, the hash table is searched and the primitive event is delivered to the address where the operation is performed with regard to the primitive event, and whether the next execution is performed or not is determined through the operation. Figure 8 shows the process to access the event query tree through the hash table when a primitive event occurs. Let us assume that a primitive event $E_{1}$ occurs. To identify the address of the event query tree where the operation related to $E_{1}$ occurs in the hash table, the hash table is searched, and the primitive event is delivered to Node 2, where the virtual operator OA(1,6) and redundant operations are performed. 

**Figure 9:**
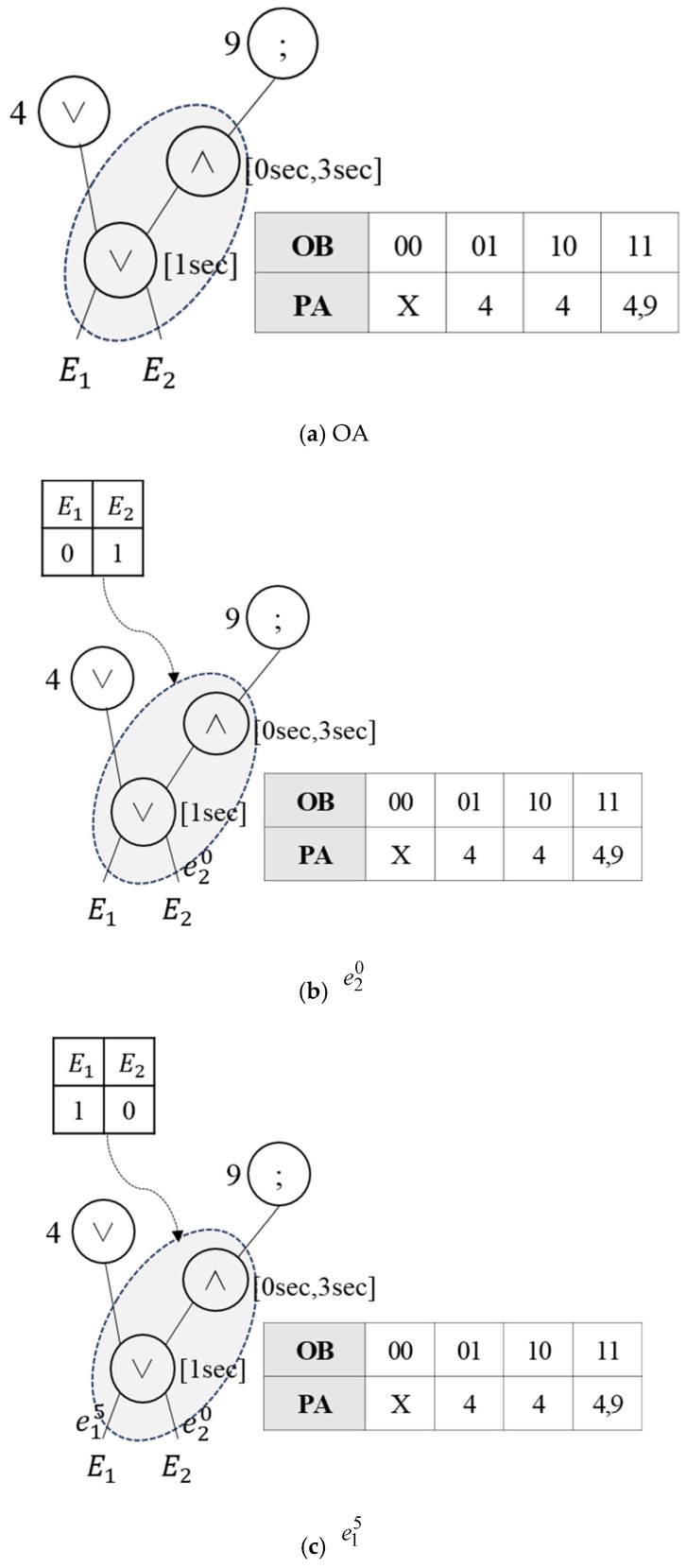

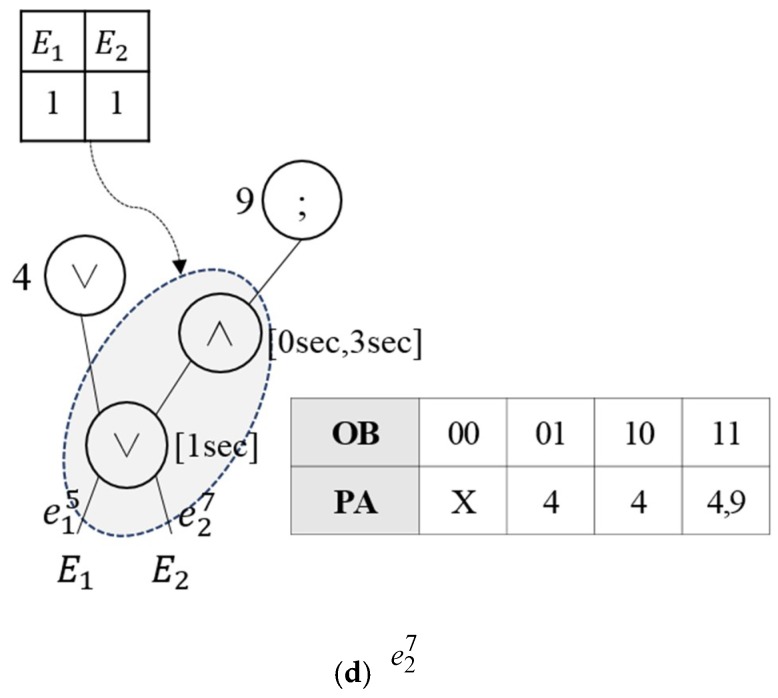
Complex event query processing procedure.

Since the similar operations are expressed as a single virtual operator, the existing RCEDA processing procedure cannot be used. Thus, the virtual operator employs the EOIT to process multiple operations, which are in similar or inclusion relationships. Figure 9 shows the complex event detection process with regard to virtual operators. Let us assume that two complex events, $Q1\;:\;WITHIN(E_{1}\vee E_{2},1\sec)$ and $Q2\;:\;WITHIN(E_{1}\wedge E_{2},0\sec,3\sec))$, are registered. The two complex events have different time attributes, but the OR and AND operations are performed with regard to primitive events $E_{1}$ and $E_{2}$. Thus, OA is created with regard to two complex events, $E_{1}$ and $E_{2}$, as shown in Figure 9a. The OR operation has a time attribute of 1 s and the AND operation has 0–3 s time attributes. Let us assume that new primitive events $e_{2}^{0}$, $e_{1}^{5}$ and $e_{2}^{7}$ occur sequentially. Here, $e_{i}^{j}$ means that event type i occurs at time j. Once the first event, $e_{2}^{0}$, occurs as shown in Figure 9b, a bit that represents the occurrence of $E_{2}$ is set to one, because $e_{2}^{0}$ occurred within 1 s. The OR operation satisfies the condition even if only a single event occurs. Thus, when the OB of the EOIT is arrayed as “01”, the next execution request is given to Parent Node 4, where the next operation will be performed. When the second event $e_{1}^{5}$ occurs as shown in Figure 9c, the occurrence of $E_{1}$ is set to one, because the OR operation that performs a process every 1 s occurred within 1 s. Since the previously occurred $e_{2}^{0}$ is outside the 0–3 s range, which is the condition of the AND operation, the occurrence of $E_{2}$ is set to zero. Thus, the OB of the collective events becomes “10”. When the OB of the EOIT is “10”, the next execution node is 10. Thus, the next execution request is given to Node 10. When the last event $e_{2}^{7}$ occurs as shown in Figure 9d, the occurrence of $E_{2}$ is set to one, because the OR operation that performs a process every 1 s occurred within 1 s. Since the previously generated $e_{1}^{5}$ occurred within 0–3 s, which is the condition of the AND operation, the occurrence of $E_{1}$ is set to one. Thus, the OB of the final event becomes “11”. The next execution request is given to Nodes 4 and 9 because both $E_{1}$ and $E_{2}$ occur. 

## 4. Performance Evaluation

To prove the superiority of the proposed method, performance evaluation was conducted compared with the existing RCEDA method [33]. For the performance evaluation environment, Intel Core i3-2100 processor and six-gigabyte memory were used, and the operating system was Microsoft Windows. The modules for CEP were implemented by Java. Arbitrarily, 5000 to 40,000 primitive events were created, and processing times were compared according to the number of events, ratio of similar operations, and ratio of redundant operations. 

**Figure 10:**
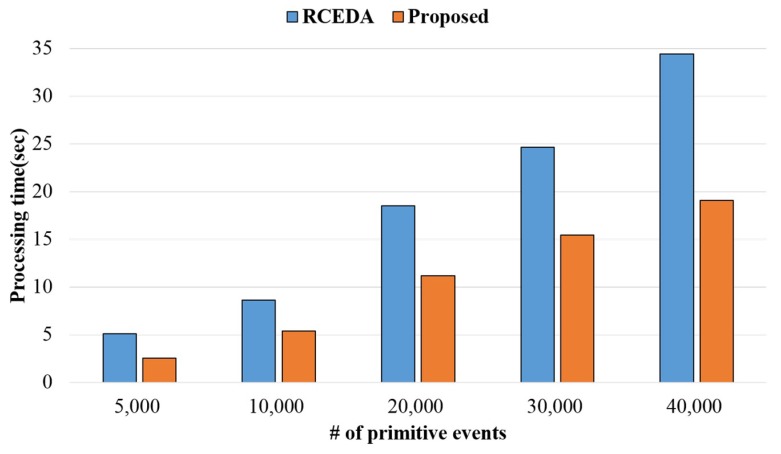
Event processing time according to the number of events.

The processing time was compared with the existing RCEDA method according to the number of primitive events through the created data. Figure 10 shows the comparison results of processing time according to the number of primitive events. The processing efficiency of the proposed method became better than that of the existing method as the number of primitive events increased, and the proposed method showed higher processing speed, by 58% on average, than that of the existing method. The existing method performed calculations of events that had similar operations individually, whereas the proposed method grouped similar operations to reduce the repeated computation, thereby exhibiting better processing efficiency than processing them individually.

**Figure 11:**
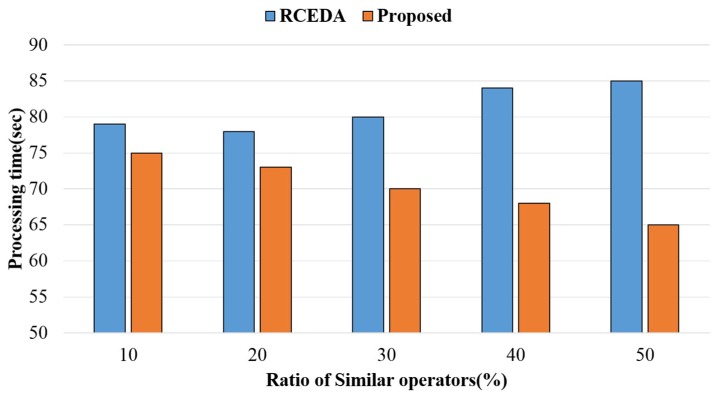
Processing time according to a ratio of similar operations.

Figure 11 shows the processing time according to the ratio of similar operations. In the experiment, approximately 40 identical complex event queries were used in the existing and proposed methods. As the ratio of similar operations increased, the existing method showed a constant processing time, whereas the proposed method exhibited a decreasing processing time. In addition, the processing performance of the proposed method was higher when the ratio of similar operations was 30% to 50% compared to when it was 10 to 20%. Since the proposed method combined and processed similar operations as a single virtual operation, which was not considered in the existing method, it reduced computational load compared to the existing method. Thus, the above results verified that the proposed method was on average 13.2% faster in processing time compared to the existing method. 

**Figure 12:**
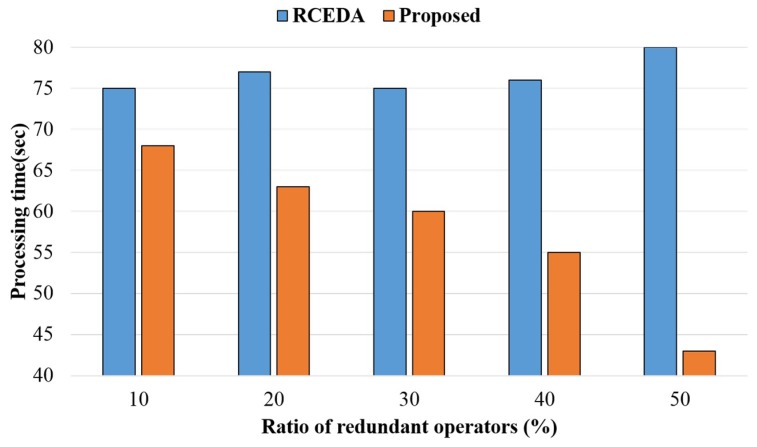
Processing time according to a ratio of redundant operations.

Figure 12 shows the comparison results of the processing time according to 10 ratios of redundant operations in the level 1 tree structure among the complex event queries, using 40,000 events. The proposed method reduced the total computation by conducting a single redundant operation and using the result in all other redundant operations, in contrast with the existing method. Thus, the proposed method had a processing time on average 18.8% faster than that of the existing method, as the ratio of redundant operations increased in the complex event queries. 

**Figure 13:**
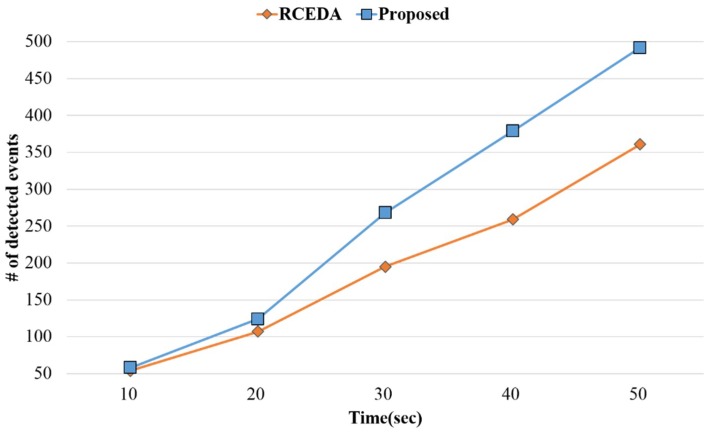
The number of detected events.

The performance evaluation was conducted to prove that the proposed method requires less computation than that of the existing method by using a virtual operator. In the performance evaluation, event detection was compared between the existing and proposed methods using virtual operators located in level 1 (except for parent nodes that had no virtual operators) using 40,000 events in the complex event query tree. Figure 13 shows the number of detected events at 10 s, 20 s, 30 s, 40 s and 50 s with regard to operations located in level 1, where the virtual operators are present. As shown in the figure, computational load was minimal between 10 and 20 s, but after 30 s, the proposed method showed much faster computation than that of the existing method.

## 5. Conclusions

In this paper, we proposed a complex event-processing method for sensor streams in the IoT environment. The basic CEP method has a problem of increasing overall computation since it processes similar and redundant operations individually with regard to common primitive events. In the proposed method, the characteristics of the operators used to perform complex events are analyzed, and similar and redundant operations are detected. Similar operations are processed as a single virtual operation, and redundant operations are combined into a single operation to process complex events. Thus, the proposed method can shorten processing time because it does not perform redundant comparison operations or inspections on the same events. The performance evaluation on processing time was conducted according to the event processing time and ratios of similar and redundant operations, and the results showed that the proposed method improved processing time compared to that of the existing method. As sensor big data is created along with expansion of IoT-based services, research is needed to process large amounts of sensor stream data. In future works, we will conduct CEP distributed processing methods for processing large sensor stream data. In addition, we will implement the proposed method for actual open CEP systems and perform performance evaluations as workloads change.

## Figures and Tables

**Table 1:** Similar operations on the same primitive events.

Similar Operation	Participated Operation		Description
OA	$E_{1}\vee E_{2}$	$E_{1}\wedge E_{2}$	When events that are contained in the OR and AND operations are the same
SS	$E_{1};E_{2}$	$E_{1};E_{3}$	When a precedent event occurs between events included in two SEQ operations
OS	$E_{1}\vee E_{2}$	$E_{1};E_{3}$	When a precedent event in the SEQ operation among the events included in the OR and SEQ operations is included in the OR operation

**Table 2:** Similar operation detection results.

Similar Operation	Participated Operation	Parent Node
OA(1,6)	$E_{1}\vee E_{2}$	4
	$E_{1}\wedge E_{2}$	9
OS(3,7)	$E_{4}\vee E_{5}$	4
	$E_{4};E_{6}$	9

**Table 3:** Redundant operation detection results.

Redundant Operation	Participated Operation	Parent Node
SO(2,8)	$E_{3};E_{1}$	4, 9
